# Response shifting: a qualitative meta-synthesis on response shift

**DOI:** 10.1007/s11136-026-04290-0

**Published:** 2026-06-06

**Authors:** Kara Schick-Makaroff, Antoinette F. Davey, Véronique Sébille, Mirjam A. G. Sprangers, Richard Sawatzky, the Response Shift –in Sync Working Group

**Affiliations:** 1 Faculty of Nursing, University of Alberta, Edmonton, Canada; 2 Mood Disorders Research Centre, University of Exeter, Exeter, UK; 3 Exeter Collaboration for Academic Primary Care, College of Medicine and Health, University of Exeter, Exeter, UK; 4 Inserm UMR 1246 Sphere “Methods in Patient-Centered Outcomes and Health Research”, Nantes University - University of Tours, Nantes, France; 5 Unit of Methodology in Clinical Research and Biostatistics, University Hospital of Nantes, Nantes, France; 6 Department of Medical Psychology, Amsterdam UMC Location University of Amsterdam, Amsterdam, The Netherlands; 7 Amsterdam Public Health, Mental Health, Amsterdam, The Netherlands; 8 School of Nursing, Trinity Western University, Langley, BC Canada; 9 Centre for Advancing Health Outcomes, Providence Health Care Research Institute, Vancouver, BC Canada; 10 Institute of Health and Care Sciences, and Centre for Person-Centered Care (GPCC), Sahlgrenska Academy, University of Gothenburg, Gothenburg, Sweden

**Keywords:** Response shift, Qualitative, Patient-reported outcomes, Systematic review, Qualitative meta-synthesis, Interviews, Focus groups

## Abstract

**Purpose:**

This qualitative meta-synthesis aimed to (1) describe health-related studies that examined response shift using qualitative methods, and (2) synthesize the qualitative results about response shift.

**Methods:**

We systematically searched MEDLINE, PSYCINFO, CINAHL, EMBASE, Social Science Citation Index, and Dissertations and Theses Global to identify health-related studies using qualitative and mixed methods designs to examine response shift (n = 2221). Findings were classified using constant targeted comparison and “imported concepts” (e.g., recalibration).

**Results:**

Of 1010 records screened, 33 had full-text screening; 14 were included, 10 of which used patient-reported outcome measures as part of their qualitative methods. Six studies specified a qualitative methodology. All of the 14 studies inferred evidence of response shift. Recalibration evidence was related to comparisons to previous health states or to others with poorer health; pre-existing expectations of current health; and adjustment of their quality of life standard. Reprioritization evidence was related to shifting life priorities to compensate for changing health status and forced changes in goals/priorities due to severity of treatment effects. Reconceptualization evidence was associated with changes in participants’ health conditions and/or treatment and commonly co-occurred with reprioritization highlighting their interconnectedness. Authors of 10 studies noted possible alternative explanations of response shift, including: recall bias, incapacity of verbalizing experiences/feelings, irrelevant stimuli, and response bias, none of which ruled out concurrent occurrence of response shift.

**Conclusion:**

Future work is needed to engage in dialogue about how multiple lenses towards inquiry and analysis may be leveraged to examine the multiplicity of ways in which people experience change in meaning.

**Supplementary Information:**

The online version contains supplementary material available at 10.1007/s11136-026-04290-0.

## Introduction

Investigation into response shift has unfolded through the use of quantitative approaches analysing the results of patient reported outcome measures (PROMs), and qualitative approaches elucidating responses to PROMs as well as employing unstandardized and open questions. The former has received the lion’s share of investigation. Most previous systematic reviews have targeted syntheses of the quantitative studies [1–6]. A smaller body of literature has leveraged qualitative or mixed methods designs to investigate response shift and unpack individual differences. Such research provides insights that might otherwise be missed. Whereas most quantitative approaches draw upon response shift methods that predominantly provide global or average information at the group-level of a population, qualitative approaches may investigate the underlying heterogeneity at an individual-level [7]. While quantitative approaches have made important contributions to the detection and measurement of response shift effects, qualitative inquiry offers a window into description and understanding of response shift [8]. Qualitative approaches offer the opportunity to inquire about changes in the meaning of subjective evaluations [8]. Further, qualitative research can contribute understanding about how response shift occurs by examining how response processes (i.e., “what people do, think, or feel when interacting with, and responding to [measurement items]” [9], p. 2] relate to corresponding changes in the meaning of the measurement scores (i.e., the scores that are produced based on people’s responses to the items) [10, 11]. Together, these potential insights gained through the use of qualitative interpretive inquiry into subjective experiences and expressions may add essential, complementary perspectives of response shift.

Schwartz et al. [12] characterized qualitative inquiry about response shift by conducting a scoping review. Of the 39 included studies, 27 used qualitative methods to investigate response shift, eight focused on advancing response shift methodology, six demonstrated response shift in relation to one or more PROMs, and four “referenced response-shift theory with or in contrast to another theory as an explanation for study findings” (p. 138). Note that each study could be categorized in more than one way. The scoping review offered a helpful descriptive categorization of qualitative response shift studies, which included focus groups, cognitive interviews, semi-structured interviews, and open-ended survey items. However, a synthesis of the *results* of qualitative research that explicitly focuses on response shift has not been published to date.

To inform future investigations and further advance the field, it is important to understand what counts as evidence for inferring the different types of response shift (recalibration, reprioritization, and reconceptualization) and whether there are any alternative explanations. We followed Sprangers and Schwartz’s [13, 14] definition that response shift “refers to a change in the meaning of one's self-evaluation of a target construct as a result of: (a) a change in the respondent's internal standards of measurement (scale recalibration, in psychometric terms); (b) a change in the respondent's values (i.e. the importance of component domains constituting the target construct); or (c) a redefinition of the target construct (i.e. reconceptualization)” (p. 1508). The aims of this qualitative meta-synthesis were therefore to: 1) describe health-related studies that examined response shift using qualitative methods, and 2) synthesize the qualitative results about response shift.

## Methods

**Fig. 1:**
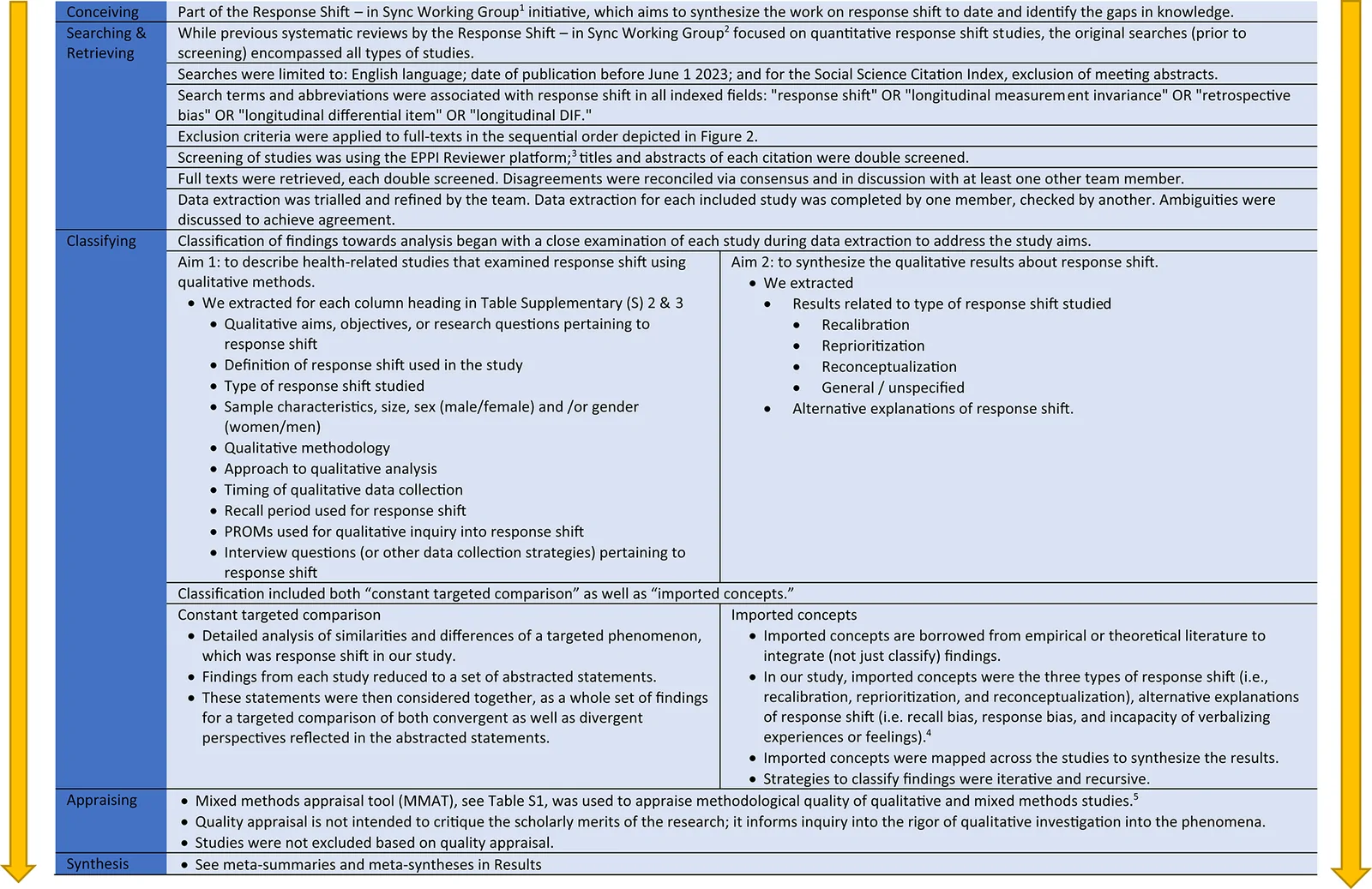
Flowchart of qualitative meta-synthesis approach. ^1^See [17, 42]. ^2^See [5, 6]. ^3^See [43]. ^4^See [7, 8]. ^5^See [44]

We followed a qualitative meta-synthesis approach[15] and used the steps outlined by Sandelowski and Barroso[16]. These steps included a) conceiving the synthesis, b) searching and retrieving literature, c) classifying findings, d) appraising findings, e) synthesizing findings into meta-summaries, and f) synthesizing findings into a meta-synthesis (see Fig. 1).

**Fig. 2:**
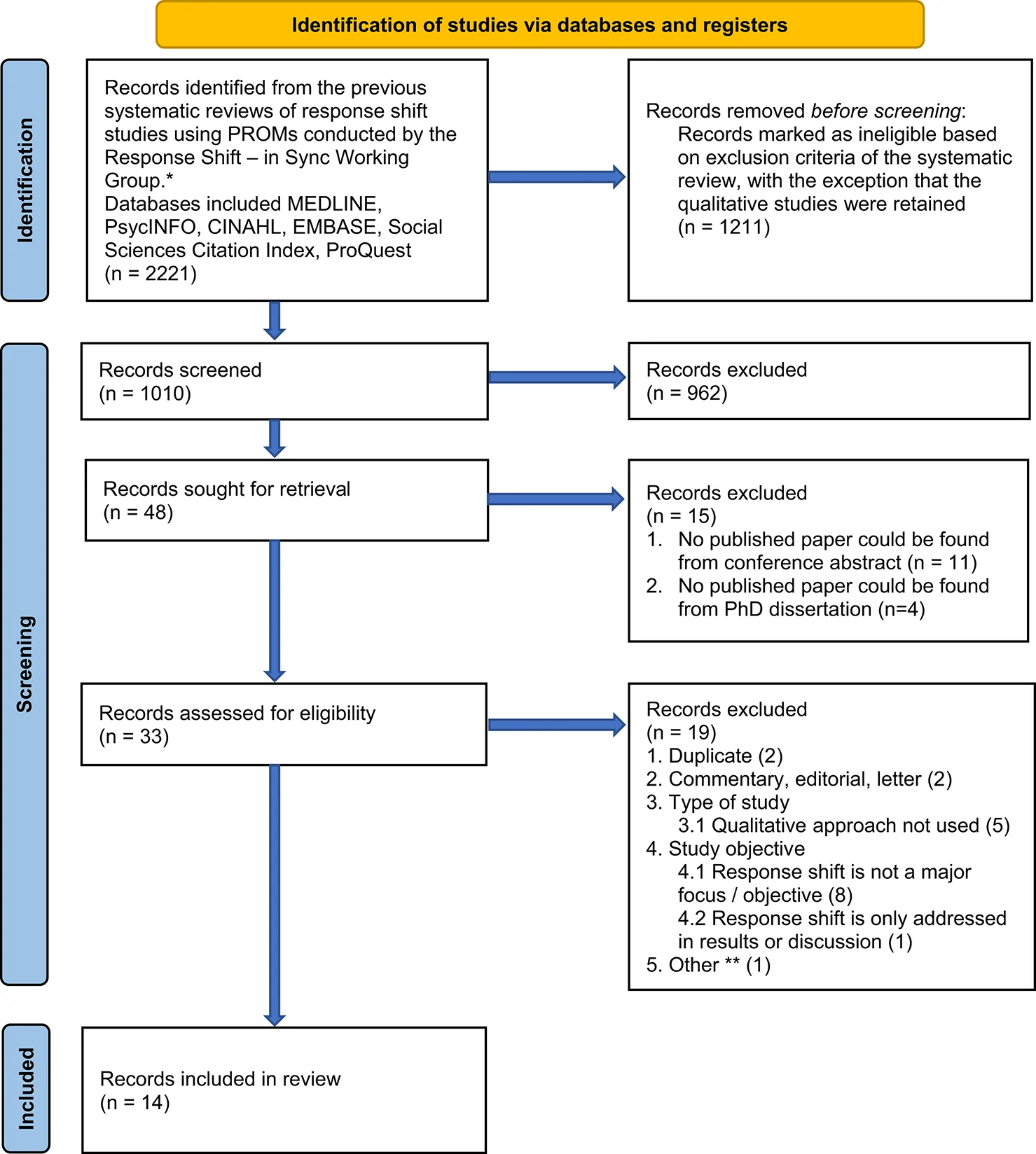
Flowchart of the review process. *See [17] and [5, 6] for details. **The objective focused on examining assumptions underlying the thentest and analyses focused on a post-then comparison. Thus, response shift was not explicitly examined even though it was a major focus

We used the search results of a systematic review of quantitative response shift studies using PROMs conducted by the Response Shift – in Sync Working Group[17]. Searched databases are included in Fig. 2 and search terms in Fig. 1. The rigor was enhanced through the following: a registered protocol; double-screening of titles, abstracts, and full-texts; disagreements reconciled via consensus through discussion; use of the EPPI Reviewer platform to facilitate systematic extraction and in-depth collaborative synthesis; and use of an established tool to support appraisal of study quality as part of the synthesis.

We sought to include all health-related studies that examined response shift using qualitative methods and where response shift was a major focus or objective. These included qualitative and mixed methods designs (where qualitative results could be isolated for extraction). We excluded studies where response shift was not a major focus/objective or was only addressed in the results or discussion.

## Results

Out of 1010 titles and abstracts screened, 33 studies went forward for full-text screening, and 14 of these were included in the final synthesis (see the PRISMA flowchart in Fig. 2). Although most of the quality criteria were met (See Table S1), 12 of the 14 studies did not articulate clear research questions (apart from the study purpose, aim or objectives) and 3 of qualitative studies lacked coherence due to unspecified analysis.

### Synthesizing findings into meta-summaries: description of studies (aim 1)

The qualitative aims, objectives, or research questions of the 14 included studies addressed response shift [18–25], respondents’ QOL definitions, appraisals, or adaptations to changing health [26], change in QOL [27, 28], cognitive processes underlying QOL appraisals [29, 30], and exploration of anchoring vignettes for measuring response shift [31]. Definitions and orientations towards response shift were informed by Sprangers and Schwartz [13, 14, 32] in 13 of the studies [18–28, 30, 31], whereas one study [29] was informed by Rapkin and Schwartz [33] and Tourangeau et al. [34] because they describe the cognitive processes underlying quality of life assessment.

Details about each study are provided in supplementary files for studies that had a PROM as part of the response shift investigation (Table S2), or not (Table S3). There was considerable variation in sample characteristics of each study, including: (a) participants’ health conditions (cancer [19, 20, 22, 25, 29], stroke [18], arthritis [21], spinal cord injury [26], psoriasis [31], HIV/AIDS [30], and oral decay [28]); (b) participants’ treatments (stem cell transplant [23], dialysis [27], rehabilitation [24]); and (c) sample sizes (20–521 participants). Sex (male/female) was reported in eight studies [18, 21, 22, 25–27, 30, 31]; gender (men/women) was reported in four other studies [19, 20, 23, 29]. Two had men only because of their focus on prostate cancer [19, 20], eight studies had approximately half of their sample as male/men [18, 22, 23, 25, 27, 29–31], one had more male (80%) than female [26], and one had more female (87%) than male [21]. Two studies did not report on sex/gender [22, 26].

**Fig. 3:**
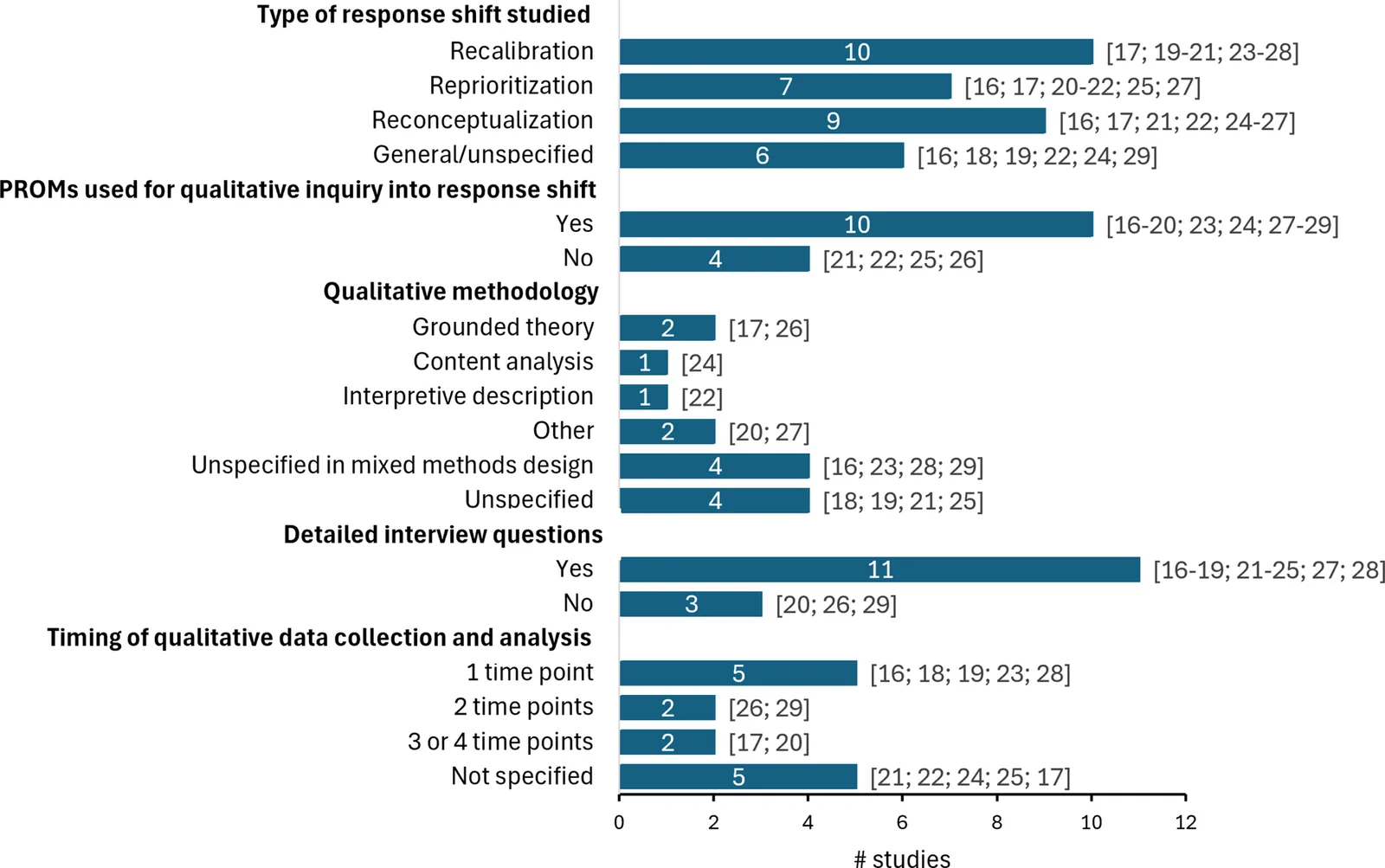
Methodological characteristics of included studies

Figure 3 provides an overview of methodological characteristics of the 14 included studies. Tables S2 and S3 provide further details. Ten studies included PROMs as part of their qualitative methods; the PROMs used are listed in Table S2. Only six of the 14 studies identified having used an established qualitative methodology (i.e., grounded theory, content analysis, interpretative description) or a unique qualitative methodology (i.e., exploratory longitudinal multiple case study [22] or a Three-Step Test Interview combining cognitive think-aloud interviewing and verbal probing techniques [29]). Four studies did not specify a qualitative methodology and four used an unspecified qualitative approach as part of mixed methods design. Six studies [19, 24, 26, 28–30] followed traditional analytic approaches often used with the chosen qualitative methodology (for example, a constant comparative analysis in a grounded theory study [19]); three studies [22, 23, 25] tailored a unique analytic approach (for example, an exploratory, longitudinal multiple-case study utilized mind maps of think aloud data and response shift explanation [22]); and 5 studies did not specify an analytic approach. Eleven studies provided detailed information about the interview questions. The timing of the qualitative data collection varied with nine studies providing a detailed outline of when qualitative data collection occurred (e.g., at the start, during, or upon completion of receiving a treatment). Recall periods varied across the 14 studies with the minimum recall period being 4 weeks and maximum 6 years.

### Synthesizing findings into a meta-synthesis: synthesis of qualitative results about response shift (aim 2)

Table 1 provides abstracted statements [16], illustrative of how response shift was identified based on the qualitative data, and organized by the type of response shift (including general or unspecified response shift) and alternative explanations of response shift. The following subsections discuss both similarities and differences [16] across the studies based on the authors’ conclusions.

**Table 1:** Qualitative results about response shift (Aim 2)

Study	Type of response shift studied	Evidence of response shift Exemplar qualitative findings that the authors inferred as evidence of response shift	Alternative explanations Exemplar qualitative findings that the authors inferred as evidence of alternative explanations of response shift
Ahmed et al. [18]	Recalibration	Not addressed.	*Recall bias*: Some patients forgot to mention changes indicating a recall bias. *Incapacity of verbalizing experiences/feelings:* Many patients communicated very little during interviews or used different words to describe the same problem at different time points. *Irrelevant stimuli:* Major life events occurred between evaluations however these were not associated with response shift.
	Reprioritization	The authors inferred evidence of reprioritization from examples of two patients who completed the Patient Generated Index (at 6 and 24 weeks) noting that they placed different values on the areas of QOL over time.	
	Reconceptualization	“For those whose semi-structured interview revealed a response shift they had either reduced (n = 6) or selected completely different (n = 7) areas between the 6- and 24-week evaluation.” (p. 2254) Of the two patient case studies (see above), only one individual changed the areas of importance for QOL.	
	General / unspecified	After reviewing 46 interviews, 13 (28%) people were classified as having expressed verbalizations reflecting a response shift.	
Beeken et al. [23]	Recalibration	“Fifteen patients (54%) indicated that they had engaged in a recalibration of what, for them, constitutes ‘good’ HRQOL. Patients described how previously they would not have evaluated their HRQOL in the same way….A number of patients justified their positive descriptions of their current HRQOL, stating that it was ‘good for their age’ or ‘considering what they’d been through” (p.157). Patients expectations were lower, which was linked to their recalibrations. The response shift described by patients involved a change in standards in their adjustment to difficulties post-haematopoietic stem cell transplant.	*Recall bias:* May have affected patient recollections. *Incapacity of verbalizing experiences/feelings*: The majority of patients did not provide clear indications regarding changes to their definition of HRQOL following illness or treatment experience.
	Reprioritization	Sixteen patients (57%) indicated “that what was important to their HRQOL was now different. Many discussed how work, money, physical appearance, and a ‘tidy’ home and garden were things that had been important prior to transplant. These became less important following the transplant….On the other hand, family and friends were seen to be more important than previously….Patients also described ‘little things’ such as being in the garden, having visits from friends or simply feeling good throughout the day as important and current priorities, rather than focusing on things they could no longer do.” (p. 157) This shift in values facilitated a change in focus from compromised QOL and towards heightened values.	
	Reconceptualization	The authors commented that it was difficult to identify evidence of reconceptualization because participants struggled to articulate how or whether QOL had changed since their illnesses or haematopoietic stem cell transplant. Further, it was challenging to disentangle reconceptualization and reprioritization.	
	General / unspecified	Not addressed.	
Elliott et al. [27]	Recalibration	“When beginning dialysis, [people living with dialysis] reported that life on dialysis was worth living and recalibrated their QOL assessments incorporating this standard into their daily lives.” (p. 1497) Patients “reported how their adjustment to dialysis included a redefinition of QOL when dialysis began—that adjustment demanded a restructuring of daily life and a recalibration in defining life and its quality. From then on, dialysis dictated their daily schedule and supported continuing life experiences.” (p. 1502) “The [people living with dialysis] comments … demonstrate their initial recalibration—redefinition—of their QOL standard to the basic insight that dialysis and its challenges were worth the life they provided.” (p. 1503)	*Incapacity of verbalizing experiences/feelings*: Patients struggled to explain their QOL responses when living with changing circumstances.
	Reprioritization	With the burden of dialysis on their bodies, “[people living with dialysis] noted that these changes also brought new insights regarding their QoL, as it moved from one of ‘Thriving’ to becoming one of ‘Surviving.’ At this point, people living with dialysis (PWDs) did not question their basic standard regarding QOL (participating in life was dependent on continuing dialysis), but their priorities became clearer: Family support and not being a burden were increasingly important.” (p. 1501) As people living with dialysis experienced declining health as the “new normal level of functioning, [their] comments revealed that they were also beginning to question continuing dialysis, because they found that their QOL assessments were not consistent with their values and priorities.” (p. 1501). With extremely limited activities and lives “the discussion of whether to continue dialysis—whether to continue life—was raised by the [PWDs] and their family members in the interviews. Their comments then named the role of QOL in this decision-making. Their priorities and values became the substance of the decision-making to evaluate whether the standard of ‘staying alive with dialysis’ would still be worth it. When the priorities and values could no longer be maintained due to the existing level of functioning, the basic reason to continue dialysis and continue life—quality of life—could not be sustained. They decided that it was time to die.” (p. 1502)	
	Reconceptualization	Thick, detailed description was provided of reconceptualization over the course of living with kidney failure and dialysis, mapped alongside participants’ changes in health. Participants first reported “thriving,” having an excellent QOL, and describing how they engaged in the creation of a “good life.” (p. 1500) As the burden of dialysis on their bodies continued, their health was changing and they described “surviving,” from fair towards poorer QOL. For participants at “end-stage: not meeting the QOL standard,” their conceptualization of QOL was at odds with their priorities for living, including questioning whether to continue with dialysis (without which, they would die). In this manner, reprioritization overtly informed their definitions of QOL (reconceptualization).	
	General / unspecified	Not addressed.	
Gregory et al. [28]	Recalibration	Patients reporting changes in the “margins of relevance of oral health” when their health deteriorated, suggesting a comparison of prior health states to their current health state.	*Response bias*: Social comparison and the general process of constructing margins of relevance have been related to response shift.
	Reprioritization	Not addressed.	
	Reconceptualization	The authors provided only one comment that participants’ changes about the relevance of dimensions of oral health were an example of reconceptualization. “Changes in the margins of relevance can be seen as beta change, (changes in internal standards) whereas changes in the relevance of dimensions can be seen as gamma change, (changes in values and reconceptualisations of quality of life).” (p. 1866)	
	General / unspecified	Not addressed.	
King et al. [24]	Recalibration	Not addressed.	Not addressed.
	Reprioritization	Patients’ diseases changed their perspective thus they reprioritized what was important to them.	
	Reconceptualization	The authors provided only one comment that goals and priorities changed. Therapists observed that participants’ perspectives of what was important, as well as their priorities, changed during and after rehabilitation. The authors noted that this reflected both reconceptualization and reprioritization.	
	General/ unspecified	Participants described observing and using concepts of response shift. The participants did not use response shift or transformative learning frameworks to describe their process of change, however they used various concepts from these frameworks such as “catalyst, antecedents, mechanisms, change in priorities and what is important.” (p. 7)	
Korfage et al. [20]	Recalibration	Not addressed.	*Irrelevant stimuli:* The dysfunctions (e.g., sexual, urinary, bowel) that patients experienced were not perceived as health problems that they considered when completing QOL PROMs. “A consistent finding in our interview data was that respondents tended not to consider urinary, bowel and especially sexual dysfunction as aspects of health. These respondents did not exclude such dysfunction because they had forgotten about it; they reported urinary, bowel and sexual problems without hesitation when asked specifically about them. They consciously excluded it from considerations regarding general health questions because they deemed it irrelevant to their ‘health’.” (p. 916)
	Reprioritization	Not addressed.	
	Reconceptualization	Not addressed.	
	General / unspecified	Many patients accepted the side effects of their treatment as inevitable consequences of being treated for their life-threatening condition prostate cancer. Authors considered examples of response shift from patients where they described getting used to dysfunctions after some time as an acceptance, and did not consider the consequences of treatment when answering questions about their health.	
Osborne et al. [21]	Recalibration	Patients found acceptance through comparing their circumstances with other people and/or events whether it was fictitious or real, which recalibrated their sense of normalcy.	*Recall bias*: Despite variability in the lengths of time between different stages of data collection, patients were confident and clear about their answers. *Response bias*: The unblinded interviews (patients were reminded of their initial answers) could not exclude reporting bias.
	Reprioritization	Not addressed.	
	Reconceptualization	Not addressed.	
	General / unspecified	The authors reported three types of response shift in their results: positive, negative and absent. Presence of response shift appeared to impact the benefits individuals perceived from taking part in self-management programs. Those who had positive response shift tended to overestimate the severity of their condition (“I now realize I was much better than I thought I was” p. 463), whereas those with negative response shift did the opposite (“I now realize that I was worse than I thought I was.” p. 463)	
Rohn et al. [26]	Recalibration	Social comparison acted as a mechanism in that patients framed their experiences and retained a sense of self by comparing themselves with others. Patients had lowered their expectations (a recalibration of standards), to avoid disappointment.	Not addressed.
	Reprioritization	Not addressed.	
	Reconceptualization	9 of 40 participants spoke of reconceptualization, which the authors described under the theme “behavior-driven response shift.” Quotes exemplified that these participants accepted they could no longer do the same things, and responded to their spinal cord injury by finding new ways to contribute, redefining social roles, having a new “job”, and feeling empowered. These participants had the highest QOL (8.28/10 on SCI-QOL).	
	General/ unspecified	The authors presented four themes within the results which included (1) behavior-driven (i.e. taking an active role in responding to spinal cord injury, (2) awareness-driven (i.e. focusing on sense of self-worth and identity), (3) social comparison (i.e. comparing circumstances to those of others), and (4) resignation and despair (i.e. being stuck or giving up). Family support and optimism featured in participants’ positive emotional and cognitive self-assessments. Additionally, participants normalized their experiences, whilst positively reframing their situations, and stating that they were “blessings.” Any complications from bladder and bowel management were seen as obstacles that were manageable. Participants appeared to accept their limitations. Participants who had little or no response shift had a sense of being stuck. The comments from most participants were on the loss of mobility and independence, however there was little or no talk of self-determined actions from participants to improve and maintain their QOL. Participants did not redefine their goals and expectations except when they had previously abandoned them. Some participants avoided social engagements as a result of their injury and/or bladder/bowel movement implying that they ignored health related or social problems.	
Serdà i Ferrer et al. [19]	Recalibration	At first, patients interviewed in this study “were not able to evaluate the severity of the impact of the new side effects because the side effects had only just appeared. The lack of experience of living with side effects therefore positively biased patients’ evaluations of QOL.” (p. 36) However, their awareness of the impact of the side effects on QOL was later realized to be present after treatment, suggesting a comparison of experiences and health status.	*Recall bias*: Patient’s current state of health could influence how they recalled their previous state of health or perception of QOL.
	Reprioritization	“The subgroup of men ages 65 to 85 reprioritized more stable dimensions such as family support and emotional state to compensate for less stable dimensions such as urinary incontinence, fatigue, or impotence to rebalance their QOL. One participant comment included: ‘My wife and kids are the most important thing in my life. They are my support. When I am tired they help me and I don’t feel so bad.’ Younger participants (ages 45 to 64) use family support and emotional state to cope with and fight against the side effects.” (p. 37–38). The authors provided the following conclusion: “stable dimensions, such as family support and emotional state, differ in importance throughout the disease process and that difference also exists between younger and older participants.” (p. 37)	
	Reconceptualization	The authors provided only one comment about reconceptualization. “The reconceptualization mechanism coincides with the end of reprioritization, when disease-related coping strategies begin to stabilize. The mechanism is characterized by satisfactory adaptation to the disease’s effects, signaling the beginning of a new post-disease period.” (p. 39)	
	General / unspecified	Not addressed.	Not addressed.
Schwartz and Rapkin [30]	Recalibration	"Qualitative explanation of ipsative scores: During assessment, individuals were probed to determine reasons for discrepant ratings on the thentest. There were 247 people whose prescore and then score differed by more than one point on the 10-point Global QOL rating (the Large-Discrepancy group). These individuals were asked to explain (a) how ‘the way that you think and feel about your overall health changed in the last 6 months, to help explain the difference in these ratings?’ Conversely, there were 272 people whose prescore and then score differed by one point or less (The No-Discrepancy group) at 6 months. These study participants were asked, ‘Even though these answers are similar, has the way that you think and feel about your overall health changed in the last 6 months?’ These open-ended responses were then coded into 13 explanatory categories. The Large-Discrepancy group had a mean of 1.38 explanatory codes at 6 months. The No- Discrepancy group had a mean of 0.60 explanatory codes at 6 months. The most prevalent explanatory codes for the Large-Discrepancy group were Health Worse, Health Better, Recalibration, Treatment-Related, Self-monitoring, Taking Better Care of Self, and Mental Health Problem. The most prevalent explanatory codes for the No-Discrepancy group were Health Worse (12%) or Health Better (15%). All other codes were reflected on average in 3% of the No-Discrepancy group utterances. Recalibration was not mentioned by any of the No-Discrepancy group members." (p. 385) "Participants’ explanations for the then-minus-pretest discrepancies were also related to changes in appraisal…The reasons with the most explanatory power were related to better current coping, better self-care, current mental health problems, comfort/contentment, and recalibration. It should also be noted that although recall did not statistically impact the correlation between then- minus-pretest and normative discrepancy scores, an explanation characterized as ‘’don’t remember/don’t know’ was substantially relevant to change in appraisal, explaining 22.5% of the variance." (p. 387)	
	Reprioritization	Not addressed.	
	Reconceptualization	Not addressed.	
	General / unspecified	Not addressed.	
Sprangers et al. [25]	Recalibration	9 of 75 patients' interviews were excluded because comments were not able to be coded by raters (n = 4) or patients could not compare their answers between their pretest and thentest (n = 5). 51/65 patients did not provide evidence of response shift; “they reported that their responses to the pretest and the thentest were equivalent. As expected, most of these patients indicated that they were unchanged physically.” (p. 145) The authors noted that some comments by patients evidenced underlying or mediating mechanisms of response shift including adaptation, downward comparison, and the role of expectations. 14/65 patients provided evidence of response shift: “11 clearly stated that some of their answers to the thentest provided a more favorable picture of their initial symptom level than their answers to the pretest. …. Conversely, 6 patients (3 of whom also belong to the former category) indicated that some of the answers to the thentest provided a less favorable picture of their initial health status than their responses to the pretest.” (p. 145) The authors explained that patient quotes indicated re-evaluation in the thentest of pretest levels of fatigue.	Not addressed.
	Reprioritization	Not addressed.	
	Reconceptualization	Not addressed.	
	General / unspecified	Not addressed.	
Taminiau-Bloem et al. [29]	Recalibration	The authors drew upon Rapkin and Schwartz[33], also making use of Tourangeau, Rips and Rasinski’s model[34], to explain how recalibration was evidenced by changes in response processes related to a shift in standards of comparison. The authors inferred evidence of standards of comparison: “The reference group used changed [sic] in 152 out of 342 comparisons of responses (44%) [over time]. In the majority of responses at baseline, patients verbalized a comparison to their own functioning prior to cancer diagnosis and treatment.” (p. 6) At follow-up patients may refer to their “functioning during the first weeks of radiotherapy, other cancer patients, expectations about future functioning, or other…people of the same age.” (p. 6)	*Incapacity of verbalizing experiences/feelings:* Some patients could not provide a definition of the target at either time point thus change of comprehension/frame of reference over time could not be examined. i.e. “Twelve patients could not provide a definition of the target construct at either baseline and/or follow-up. For these items, we could not examine whether comprehension/frame of reference was similar or rather changed over time. Therefore, (dis)similarity in this cognitive component could be assessed for 330 out of 342 (96%) comparisons of responses over time.” (p. 5) *Inconsistent reporting and response selection*: This cognitive process changed for 141 of the 342 (41%) of the comparisons.
	Reprioritization	The authors explained how reprioritization was evidenced by changes in response processes related to both sampling experience, and combinatory algorithm (or relative salience). [33, 34] The reported findings were inferred as evidence of retrieval / sampling strategy in all 342 comparisons of responses over time. (Dis)similarity for retrieval / sampling strategy was originally defined in the strictest sense, i.e. when the content of the samples differed over time. However, since it is unlikely that patients retrieve the exact same experiences during the baseline and follow-up assessment, authors re-assessed dissimilarity in this cognitive component by not focusing on change in the content of the samples used, but rather on the concept the samples stem from. Thus, response processes were deemed similar over time when they came from the same concept, e.g., pain as a result of cancer treatment. “Based on concept instead of content of the samples used, patients’ sampling strategy changed in 246 out of 342 comparisons of responses (72%).” (p. 6) The cognitive process ‘combinatory algorithm’ describes how respondents prioritize and combine positive and negative samples, where they can emphasize either the positive or negative experiences, or find a balance between both in arriving at an answer. “Patients were found to retrieve positive and negative samples in 220 responses at both baseline and follow-up, resulting in 220 comparisons of responses over time. The prioritization and combination of retrieved samples changed in 113 out of 220 comparisons of responses over time (51%).” (p.7) The authors also noted the co-occurrence of types of response shift. “In the majority of the comparisons of responses, we found changes in the content of multiple cognitive processes underlying one item, for example by reconceptualization and reprioritization.” (p. 9)	
	Reconceptualization	Reconceptualization was evidenced by changes in the response processes related to change in frame of reference (188 of 330 responses) by which patients re-defined their QOL. The authors also noted the co-occurrence of types of response shift. “Our results support this interconnection, since in the majority of the comparisons of responses, we found changes in the content of multiple cognitive processes underlying one item, for example by reconceptualization and reprioritization.” (p. 9)	
	General / unspecified	Not addressed.	
Topp et al. [31]	Recalibration	Not addressed.	*Incapacity of verbalizing experiences/feelings:* Verbalized explanations may not fully reflect decision processes that occur unconsciously. There is a high degree of uncertainty around whether or not verbalizations represent underlying differences in reasoning and reference frame.
	Reprioritization	Not addressed.	
	Reconceptualization	Not addressed.	
	General / unspecified	The ratings participants provided of anchoring vignettes fluctuated “non-directionally” over time and the think-aloud method did not provide an understanding as to the reasons for these fluctuations. Although ratings provided for vignettes differed between time points, there was no apparent direction of change for participants. The authors concluded that vignettes may not be an appropriate method to explore response shift with the psoriasis or multiple sclerosis population.	
Westerman et al. [22]	Recalibration	15/24 patients with small-cell lung cancer showed discrepancies between their numeric EORTC QLQ-C30 fatigue score and what they reported in interviews. The authors inferred evidence of recalibration for patients with and without discrepancies that occurred in different response strategies, including adopting a more positive perspective. Patients who had “reported to have changed their reference point after T1 (i.e. recalibration in contrast to T1) … compared their fatigue at the second and following interviews with that of other patients” (p. 859) who were worse off or other time points where they were more ill. “Another one spoke about a shift of limits which also suggests recalibration: ‘I already told you that I would change my standards.’ “ (p. 859)	*Recall bias*: Patients had difficulty remembering either the previous measurement point and/or their fatigue at that time. *Response bias:* Self-presentation was found to be an additional (coping) mechanism underlying the discrepancies found in the results.
	Reprioritization	Reprioritization occurred in only one instance, “The only exception was Ann who made a distinction between being physically and mentally tired…(i.e., changes in the importance attached to mental fatigue over time).” (p. 859)	
	Reconceptualization	Did not find evidence of reconceptualization.	
	General / unspecified	Not addressed.	

EORTC QLQ-C30, European organization for research and treatment of cancer – Quality of life questionnaire 30; HRQOL, Health related quality of life; PGI, Patient generated index; QOL, Quality of life; SCI-QOL, Spinal cord injury quality of life; SF12, Short form 12.

#### Recalibration

Of the 14 studies, ten reported findings that the authors inferred as evidence of recalibration [19, 21–23, 25–30]. Overall, recalibration evidence was related to comparisons to previous health states or to others with poorer health; pre-existing expectations of current health; and adjustment of their quality of life standard.

When patients compared their current internal standard by which they self-evaluate their health state with that of a previous time point within the study period [23, 28], including description of the impact of an intervention (e.g., chemotherapy, dialysis) on their symptom severity [19, 22, 27]. Patients often compared their QOL with others with poorer health (whether that was known or imagined) [21, 22, 26]. Many patients had pre-existing expectations of their QOL and lowered these expectations [23, 26] to not be disappointed if their health or life circumstances had not dramatically improved. Expectations were linked to the adjustments patients had to make due to the impact of interventions [22, 23, 27]. Adjustments to their QOL standard incorporated into their daily living were necessary given the increased difficulties experienced during and post treatment. For example, dialysis patients experienced their treatment as both a saviour and a life barrier as their daily lives revolved around their dialysis schedule [27]. And in a study with people living with cancer, Taminiau-Bloem et al. [29] drew upon Rapkin and Schwartz [33] to explain how recalibration was evidenced by changes in response processes related to a shift in standards of comparison. They described (dis)similarity in the cognitive process and found evidence that the comparison standard had changed over time in a considerable number of cases.

Two studies examined recalibration by comparing the thentest to the baseline pretest [25, 30]. A thentest entails the following: “At the posttest session, the participants fill out the self-report measure twice. First they are asked to report how they perceive themselves at present (conventional posttest), then they are asked to provide a renewed judgment about their pretreatment level of functioning (thentest). By taking the posttest and thentest in close proximity, it is assumed that these measures will be completed with respect to the same internal standard of measurement” [25] (p. 138). Sprangers et al. [25] found that 51/65 patients with cancer did not provide evidence of response shift, primarily due to no physical change; 14/65 patients provided evidence of response shift due to re-evaluation in the thentest; and for 9/65 patients their data were excluded either because these patients could not compare their responses between their thentest and pretest (n = 5) or the researchers were unable to code their comments (n = 4). Schwartz and Rapkin [30] found 247/521 people with HIV/AIDS had a pretest score and a thentest score that differed by more than one point on the 10-point Global QOL rating at 6 months. Based on interviews with these 247 people, the most explanatory codes for this discrepancy were “Health Worse, Health Better, Recalibration, Treatment-Related, Self-monitoring, Taking Better Care of Self, and Mental Health Problem Then” (p. 385).

One distinct finding was noted when authors inferred evidence of recalibration: dialysis patients adjusting to their dialysis schedule required recalibration associated with a shift in how they defined both life and QOL [27]. As treatment progressed and their circumstances changed, patients’ shifts in their reprioritization and reconceptualization of values led to the need to recalibrate their QOL standard. This interconnection of recalibration resulting from a shift in reprioritization and reconceptualization was not mentioned in other studies.

#### Reprioritization

There were seven studies where authors inferred evidence of reprioritization based on shifting life priorities (such as family, emotional support, finances) to compensate for changing health status, or forced changes in goals/priorities due to severity of treatment effects [18, 19, 22–24, 27, 29]. For example, family and friend support became more important and materialistic values, such as money or physical appearance, became less important [19, 23, 27]. Serdà i Ferrer et al. [19] noted that adaptation to the disease led to coping strategies that involved reprioritizing side effects and initiating a care plan to resolve them. The effects of the treatment or severity of patients’ health conditions sometimes forced patients to reprioritize their goals or priorities [22, 24]. Problems perceived prior to health deterioration or commencement of treatment were later seen as unimportant [24] and reprioritizing of symptoms assisted patients in accepting the seriousness of their illness in later evaluations [22]. Ahmed et al. [18] inferred evidence of reprioritization from examples of two patients who completed the Patient Generated Index (at 6 and 24 weeks) noting that they gave different values to the areas of QOL at these time points.

The findings by Serdà i Ferrer et al. [19] suggested that reprioritization may be associated with age. Older men (aged 65 to 85) reprioritized more stable dimensions (e.g., family support, emotional state) in order to compensate for less stable dimensions (e.g., impotence, incontinence) to rebalance their QOL, whereas younger men used these same stable dimensions “to cope with and fight against the side effects” (p. 38). Taminiau-Bloem et al. [29] explained how reprioritization was evidenced by changes in cognitive processes related to both sampling experience and combinatory algorithm (or relative salience) as per the appraisal theory by Rapkin and Schwartz [33]. They found retrieval/sampling strategy in all comparisons of responses over time and found that “patients’ sampling strategy changed in 246 out of 342 comparisons of responses (72%)” (p. 6). They also found that patients’ combinatory algorithm changed in 113 out of 220 comparisons of responses over time (51%).

#### Reconceptualization

Nine studies reported findings that the authors inferred as evidence of reconceptualization, of which eight concluded that response shift had occurred [18, 19, 23, 24, 26–29] and one concluded it had not [22]. Overall, reconceptualization evidence was associated with changes in participants’ health conditions and/or treatment. However, the QOL aspects that were reconceptualized were rarely explicated by the authors. Notable exceptions include the study by Elliott et al. [27], who provided thick, rich descriptive quotes from participants about reconceptualization, the study by Taminiau-Bloem et al. [29] who provided examples of dissimilarities in cognitive processes related to comprehension or frame of reference by which patients re-defined their QOL (which changed between baseline and follow-up in 188 out of 330 comparisons of responses), and the study by Rohn et al. [26], where 9 of 40 participants spoke of reconceptualization, which the authors described under the theme “behavior-driven response shift.” The authors of all other studies expressed the challenge to identify evidence of reconceptualization, despite having concluded that reconceptualization had occurred. For example, Beeken et al. [23] noted that participants struggled to articulate how or whether QOL had changed since their illnesses or haematopoietic stem cell transplant.

Six studies explicitly linked reconceptualization with reprioritization. While Beeken et al. [23] wrote that it was difficult to disentangle reconceptualization and reprioritization; Ahmed et al. [18] noted the two were interconnected; Serdà i Ferrer et al. [19] asserted reconceptualization was a mechanism that occurred at the end of reprioritization; Elliott et al. [27] conversely saw reprioritization informing definitions of QOL (reconceptualization); and King et al. [24] provided data that they believed simultaneously exemplified both types of response shift. Taminiau-Bloem et al. [29] further exemplified how reprioritization and reconceptualization could be disentangled even in situations where they co-occur: “In the majority of the comparisons of responses, we found changes in the content of multiple cognitive processes underlying one item, for example both reconceptualization and reprioritization” (p. 9).

#### General/unspecified response shift

There were six studies that provided findings from which the authors inferred evidence of general or unspecified response shift while not explicitly relating this to a particular type of response shift [18, 20, 21, 24, 26, 31]. The authors inferred evidence of response shift that occurred when patients described an acceptance of the consequences of treatment for their health and the inevitability of side effects from these treatments [20, 26]. There was also an acceptance that their health condition introduced limitations in their daily lives, which was described as positive reframing [26]. Osborne et al. [21] categorized response shift as positive, negative, or no response shift. Patients who had negative response shift underrated their condition at baseline, thus underestimating the negative effects of treatment, whilst the opposite occurred for those with positive response shift. Those with no response shift were assumed to represent a treatment effect that did not require adjustment. Ahmed et al. [18] and King et al. [24] stated that participants expressed verbalisations of response shift but did not further specify the type of response shift. Based on their use of vignette ratings, Topp et al. [31] were unable to find conclusive evidence of response shift having occurred.

#### Alternative explanations

Authors of 10 studies pointed to the possibility of alternative explanations of response shift, including: recall bias [18, 19, 21–23], incapacity of verbalizing experiences/feelings [18, 23, 27, 29, 31], irrelevant stimuli [18, 20], and response bias [21, 22, 28]. As one example, Korfage et al. [20] identified aspects of health, notably symptoms (e.g., urinary, bowel and sexual dysfunction), that participants considered to be irrelevant to their health. These symptoms were not considered health problems to be reported when completing QOL PROMs. These alternative explanations did not predominate or rule out the occurrence of response shift.

## Discussion

In conclusion, we found 14 qualitative or mixed methods studies that inferred evidence of response shift. Across the studies, recalibration response shift was related to comparisons to previous health states or to others who were more ill, pre-existing expectations of current health, or adjustment of respondents’ QOL standards incorporated into their daily living. Reprioritization evidence was related to shifting life priorities to compensate for changing health status and circumstances, and forced changes in goals/priorities due to severity of treatment effects. Reconceptualization evidence was associated with changes in participants’ health conditions and/or treatment and commonly co-occurred with reprioritization highlighting their interconnectedness. The authors of 10 studies pointed to possible alternative explanations of response shift, including: recall bias, incapacity of verbalizing experiences/feelings, irrelevant stimuli, and response bias, none of which ruled out the concurrent occurrence of response shift [18–23, 27–29, 31]. These findings contribute to an understanding of what counts as evidence for inferring response shift, and which alternative explanations may play a role. In this discussion, we address how these findings (description and synthesis of studies) contribute to the larger field of response shift.

### Description of the studies

Given that definitions of response shift in QOL began in the late 1990’s, we were surprised by how few qualitative and mixed methods studies (n = 14) explicitly addressed response shift in their purpose, aim, or objectives. Although the aforementioned scoping review identified more studies [12], our synthesis required stricter screening criteria resulting in fewer included studies (e.g., studies where response shift was not a major focus/objective or was only addressed in the results or discussion were excluded; see Fig. 1). All our included studies defined and drew on response shift theory. However, there is ambiguity about whether authors viewed response shift as a phenomenon to be discovered (e.g., based on a positivist epistemology) or as a construct that is created to facilitate interpretation and sense-making of observed changes in QOL assessment (e.g., based on a constructivist epistemology), which are different philosophical approaches towards qualitative inquiry of response shift. Articulation of epistemological assumptions are a tenant of qualitative inquiry [35]. Nevertheless, the pioneering work by the authors of these 14 studies took the conceptualization of response shift, which had been predominantly investigated from a quantitative point of view, and began to narratively explore response shift. Their unique orientations and findings lend themselves to different approaches towards inquiry into response shift, all of which are needed to advance the field.

Philosophical and theoretical positions provide sturdy underpinnings for qualitative and mixed methods designs, as well as chosen analytic methods [36], all of which are needed to further deepen the field. Within the included studies, there was heterogeneity with regards to their guiding qualitative methodology (explicit in 6/14) and approaches to analysis (explicit in 9/14—see Tables S2 and S3). While heterogeneity of approaches is to be expected, it is important that authors become explicit about their philosophical, theoretical, methodological and analytic frames, which are critical for rigorous qualitative and mixed methods studies [36]. Future research is needed to narratively explore aspects of response shift qualitatively and conduct translational forms of inquiry. This call is echoed by McClimans [37], and we see early examples of such translational work by Kwon et al. [38, 39] on how to use response shift in clinical encounters.

### Synthesis of qualitative results about response shift

The results regarding the reasons for why response shift occurs are consistent with the premise of adaptation being integral to the theoretical underpinnings of response shift [32]. However, the challenge to disentangle the different types of response shift raises questions about whether recalibration, reprioritization and reconceptualization can occur in isolation of one another, or whether they are truly interconnected. Although statistical methods have been proposed to investigate different types of response shift, the existing methods do not elucidate how they may be interconnected or narratively described. This raises the question of whether response shift should also be more generally assessed as a change in the meaning of how people think about and narratively express their QOL. Further considerations may be about how statistical operationalizations of response shift reflect narrative expressions of the ways in which people experience change in meaning. For example, a mixed methods examination of assumptions underlying quantitative operationalizations and qualitative representations of changes in meaning would be a significant contribution to the field. Notably, our meta-synthesis results point to the importance of carefully considering the possibility of alternative explanations.

### Strengths and limitations

Our study followed a rigour qualitative meta-synthesis approach, moving beyond description towards facilitation of in-depth understanding of heterogeneity in different types of response shift at an individual-level [7] (see Tables S2 and S3). We leveraged prior work by the Response Shift – in Sync Working Group by using search results of their systematic review of response shift effects in quantitative studies [17]. However, this study was not without limitations. Although qualitative studies were not excluded in the original searches, it is possible that some qualitative studies may have been missed. To mitigate this, we reviewed the included articles by Schwartz et al.’s scoping review [12], all of which were already identified in our original search. Furthermore, it was challenging to infer evidence of response shift from the authors’ stated findings. While some authors pointed to the challenge of identifying the different types of response shift, they also highlighted the challenges that people with lived experience had articulating the types of response shift.

This meta-synthesis reveals that multiple qualitative approaches are complementary to quantitative approaches and necessary to advance our understanding of response shift. Looking to the future, there is need for in-depth and rigorous qualitative and mixed methods research to further advance the field of response shift. This includes determining how interview guides and probes may be used to explore how diverse people experience, think about, and narratively describe a change in the meaning of quality of life. For example, realist evaluation and grounded approaches may be particularly suited to fostering further theoretical developments [40, 41].

## Conclusion

Qualitative and mixed methods studies provide in-depth insights into the occurrence of response shift. While statistical methods offer one way to examine distinct types of response shift, they do not reveal how the different response shift types may be interconnected or narratively described. This meta-synthesis reveals that multiple methodological approaches offer triangulation of evidence from different studies that may be complementary and perhaps necessary to advance our understanding of response shift.

## Supplementary Information

Below is the link to the electronic supplementary material.

Supplementary file1 (DOCX 50 kb)

## Data Availability

Not applicable.

## Code availability

Qualitative Coding Tool for EPPI Reviewer provided as supplementary material.
