# Supplementary material for: Response shifting: a qualitative meta-synthesis on response shift
Source: Qual Life Res. 2026 Jun 6;35(7):178. doi: 10.1007/s11136-026-04290-0 (PMC13242470; doi:10.1007/s11136-026-04290-0)
Supplement: Supplementary file 1 — Supplementary file1 (DOCX 50 kb) [file 11136_2026_4290_MOESM1_ESM.docx]

# Supplementary Files

S1: Qualitative Coding Tool for EPPI Reviewer

Table S1: Mixed Methods Appraisal Tool (MMAT), version 2018

Table S2: Methods: Description of qualitative response shift studies where a PROM was part of the response shift investigation (Aim 1)

Table S3: Methods: Description of qualitative response shift studies when response shift was investigated with qualitative methods (Aim 1)

## S1: Qualitative Coding Tool for EPPI Reviewer

### Aim 1 & 2

- Article
  - Author
  - Year

- Aspect / type of response shift studied

*Tag the corresponding text that describes what aspect of response shift (if applicable) the study aims to study. This may include recalibration, reconceptualisation, and/or reprioritisation. However, if the study authors do not clearly state these aspects then code the text “General/unspecified response shift”.*

- - Recalibration

*Use this code if the authors specifically state that they are studying recalibration.*

- - Reconceptualisation

*Use this code if the authors specifically state that they are studying reconceptualisation.*

- - Reprioritisation

*Use this code if the authors specifically state that they are studying reprioritisation.*

- - General / unspecified response shift

*Use this code if the authors are not clear in terms of which aspect of response shift has been studied.*

- - Other

*Use this code if the study authors have stated they are studying response shift but have not named it specifically, or if any of the previous codes do not fit.*

### Aim 1

- Qualitative aims, objectives or research questions pertaining to response shift

*Tag the corresponding text to this code: the aims, objectives or research questions of the study that are qualitatively based and only focuses on exploring response shift.* *This may include longitudinal measurement validation IF the aim of the qualitative inquiry is response shift*

- Definition of response shift used in the study
  - Yes

*Tag the corresponding text to this code that provides the definition of response shift used for the study, including the reference. However, if no definition is provided, use the following:*

- - Not stated
  - Ambiguous

- Sample characteristics and size
  - Health condition
  - Cancer
  - Mental Health
  - Orthopedic
  - Stroke
  - Other

List other diseases in "Info" box

- - No

*Tag text to this code to describe non-disease-specific sample, if applicable.*

- - Unknown

*Use this code if health-condition specific investigation is 'unspecified', 'unclear', or is otherwise not possible to determine with certainty based on information in the article*

- - Study sample size
    - Yes

*Tag the corresponding text to this code. Record a summary of the qualitative sample size information in the "Info" box. For example, if it is a mixed methods study only note down how many participants were involved the qualitative part of the study*

- - - Unknown

*Use this code if study sample size is 'unspecified', 'unclear', or is otherwise not possible to determine with certainty based on information in the article.*

- Qualitative methodology
  - Grounded theory
  - Phenomenology
  - Thematic analysis
  - ~~I~~nterpretive description
  - Descriptive exploratory
  - Content analysis
  - Qualitative approach (unspecified) in mixed methods study
  - Other

*List other qualitative methodology in "Info" box*

- - Unspecified

- Approach to analysis
  - Thematic analysis
  - Content analysis
  - Interpretative phenomenological analysis
  - Framework analysis
  - Constant comparative analysis
  - Other

*Add information in the “Info box”*

- - Unspecified

- Timing of qualitative data collection
  - Yes

*Tag the corresponding text to this code. Highlight text that describes any timing related to qualitative data collection. For example, if the study conducts multiple interviews at different timepoints, tag text that describes these timepoints. Add information in the “Info box” if needed.*

- - Unspecified

- Recall period used for response shift
  - now
  - 24 hours
  - 1 week
  - Between 1-2 weeks
  - Between 2-3 weeks
  - 1 month
  - Longer than a month
  - Other or multiple timepoints
  - Unspecified

- PROMs used for qualitative inquiry into response shift

*Tag the corresponding text to this code. Only highlight PROMs that were used in conjunction with the qualitative study. For example, if the study collected 5 different PROMs but only one was used in the qualitative part of the study then only highlight that PROM.*

- Generic m*easures of a health/QOL that can be applied to any population irrespective of disease. This includes single-time generic measure (each self-rated health status).*
  - WHOQOL-Bref
  - SIP (Sickness Impact Profile)
  - SF-6D
  - SF-36
  - SF-20
  - SF-12
  - Ryff Psychological Well-Being scale
  - RSCL (Rotterdam Symptom Checklist)
  - CES-D
  - EQ-5D
  - EQ5D VAS
  - Global QOL 10-point scale
  - Global QOL measure (single item, not further specified)
  - Cantril's Ladder
  - Health Utilities Index (HUI)
  - PROMIS-10
  - PedQL
  - QOLI-20 (Lehman 20 item QOL interview)
  - Satisfaction With Life Scale (SWLS)
  - Self-rated health 5 point scale
  - Kidscreen QOL questionnaire
  - LQOLP (Lancashire Quality of Life Profile)
  - QOL Appraisal Profile
  - Questionnaire on Life Satisfaction
  - LiSAT-11
  - LiSat-whole *(Overall question of the Life Satisfaction Questionnaire)*
  - VAS
  - Global Rating of Change (GRC, single item)
  - Unnamed self-rated health item
  - MusiQOL
  - OQ-45 (Outcome Questionnaire-45)
  - PDDS (Patient-Derived Disease Steps)
  - Unnamed life values questionnaire
  - Affective Valence of Future Time Perspective Scale
  - Other generic

*Tag text to this code, if applicable*

- - - Disease-specific m*easures developed for a particular disease-specific population.*
    - ADPI (Audiological disabilities preference index)
    - BDI
    - BDI-II
       *Tag text to this code*
    - WOMAC (Western Ontario and McMaster Universities Osteoarthritis Index
    - EORTC QLQ-BR23
    - EORTC QLQ-C30
    - SNOT-20
    - SIS (Stroke Impact Scale)
    - WORC (Western Ontario Rotator Cuff index
    - SQRQ (St George's Respiratory Questionnaire)
    - QLQ-BR23
    - QLQ-C30
    - QLQ-CL13
    - FACIT-Fatigue
    - FACT-G
    - FACT-P
    - MOS HIV Health Survey
    - FAAM-Sport (Foot and Ankle Ability Measure)
    - GDS-15 (Geriatric Depression Scale)
    - GHABP
    - MSSE (MS Self-efficacy)
    - MMSE (Mini Mental State Exam)
    - OHIP EDENT (Oral Health Impact Profile for edentulous patients)
    - PPP (Prostate Cancer Patient and Partner Questionnaire)
    - Satisfaction with knee surgery
    - ODI (spine disorders and low back pain)
    - OM-6 (Otis Media Quality of Life)
    - PCI (Prostate Cancer Index)
    - PAID (Problem Areas in Diabetes)
    - IBDQ (Inflammatory Bowel Disease Questionnaire)
    - IPSS (International Prostate Symptom Score)
    - KOOS
    - GIQLI (Gastrointestinal Quality of Life Index)
    - QOLIE-31 (Quality of Life in Epilepsy Inventory-31)
    - KSCRS (Knee Society clinical rating scale)
    - SAQ (Seattle Angina Questionnaire)
    - Anxiety and Depression Scale
    - ASES (American Shoulder and Elbow Surgeons score)
    - CDLQI (Children's Dermatology Life Quality Index)
    - CRQ (Chronic Respiratory Questionnaire)
    - DHEQ (Dentine Hypersensitivity Experience Questionnaire)
    - MacNew
    - Oral health measure (best-worst, 0 to 10), not named
    - PBSI (Preference Based Stroke Index)
    - QOLCE-55 (Quality of Life in Childhood Epilepsy-55)
    - Unnamed oral health-related QOL questionnaire
    - ZQC (lumbar spinal stenosis)
    - PAQLQ (Pediatric Asthma Quality of Life Questionnaire)
    - MSWS (Multiple Sclerosis Walking Scale)
    - IKDC form (International Knee Documentation Committee)
    - Lysholm score
    - NewMAc Health Disease Health-Related QOL Instrument
    - Other disease-specific
    - Individualized
    - SEIQoL-DW
    - Ryff Happiness Scale
    - Patient Generated Index (PGI)
    - Other individualized QOL measure (not named)
- Other
   *Instruments that measure a particular domain of health/QOL and that may or may not be applicable to any population. Also include unknown, time tradeoff, and vignettes. If vignette, choose the instrument linked to vignettes and add notes in “info” box; also select vignettes in the code “data collection strategies.”*
- Positive and Negative Affect Schedule (PANAS)
- Unknown
   *Tag text to this code. Use this code if measurement instrument is 'not specified', 'unclear', or is otherwise not possible to determine with certainty based on information in the article.*
   *Note: This code should only be used if there is no information about the measurement instrument at all. If there is any information about the instrument, create a code with a general description (e.g., "single item HRQOL measure, not named"*
- TTO (Time trade-off)
- SRI (Satisfaction and Recovery Index)
- SKIPP (St Christopher’s Index of Patient Priorities)
- MFI-20 (Multidimensional Fatigue Index)
- MFI-20
- Measure of subjective age (unnamed)
- Pain Scale (0-10)
- Pain VAS
- Pain Scale (4 point)
- GAD-2 (Generalized Anxiety Disorder Questionnaire-2)
- Fatigue Symptom Inventory (FSI)
- Fatigue scale (numeric, 11 points)
- Family Strain Questionnaire Short Form (FSQ-SF)
- GOSS (Goal-oriented subjective status)
- Caregiver Quality of Life (CQOL)
- ESS (Epworth Sleepiness Scale)
- NeuroQOL
- MAF (Multidimensional Assessment of Fatigue)
- Parent Ladder
- PHQ-2 (Patient Health Questionnaire-2)
- PHQ-4 (Sum of GAD-2 and PHQ-2)
- PHQ-9
- Prolonged Grief 12 (PG 12)
- POCA (Parent Observation of Children's Activities)
- Performance status
- K10 (Kessler 10 Scale)
- FABQ (Fear-Avoidance Beliefs Questionnaire)
- ICECAP-O (ICEpop CAPability instrument for older people)
- WSAS (Work and Social Adjustment Scale)
- MFI-6
- SAFE (Social, Attitudinal, Familial, and Environmental Acculturative Stress Scale)
- 28-item QOL measure (not named)
- BSS (Beck Scale for Suicide Ideation)
- CORE-OM (Clinical Outcomes in Routine Evaluation - Outcome Measure)
- DBAS (Dysfunctional Beliefs and Attitudes about Sleep Scale)
- Emanuel and Emanuel Medical Directive (patient preferences)
- Disablement in the Physically Active scale
- FES (Pain questionnaire)
- ISI (Insomnia Severity Index)
- ISR (ICD-10 Symptom Rating scale)
- Pittsburgh Sleep Quality Index (PSQI) (General Sleep Quality)
- The performance scales (Multiple Sclerosis)
  - - Unclassified
    - EPIC (Prostate cancer-specific HRQoL)
    - CHIME (Comprehensive Inventory of Mindfulness Experiences)
    - CPQ-RSF:8 (S*hort form of the Child Perception Questionnaire)*
    - HEI-Q Perspective Questionnaire (Health Education Impact Questionnaire) Perspective
    - CPQ-ISF:8
    - Single item
    - DCSQ (Demand Control Support Questionnaire)
    - Other

*Tag text to this code*

- No PROM used
- Interview questions (or other data collection strategies) pertaining to response shift

*Actual interview questions related to the PROMs or response shift in general should be put in the “Info box”. Any other data collection strategies (e.g., vignettes) pertaining to response shift should be coded using the drop-down lists:*

- - Interviews
  - Focus groups
  - Vignettes
  - Journaling
  - Unspecified
  - Other

*Add information in the “Info box”*

### Aim 2

- Results related to response shift

*Tag the corresponding text which clearly demonstrates the the aspect / type of response shift studied.*

- - Recalibration

*Tag the corresponding text to this code.*

- - Reconceptualisation

*Tag the corresponding text to this code.*

- - Reprioritisation

*Tag the corresponding text to this code.*

- - General / unspecified response shift

*If authors do not make a distinction among the 3 Rs, then refer to general / unspecified RS. Tag the corresponding text to this code.*

- - Other

*Use this code if the study authors have stated they are studying response shift but have not named it specifically, or if any of the previous codes do not fit. Tag the corresponding text to this code.*

- Alternative explanations of response shift
  - Recall bias
  - Response bias

*Code any text that provides explanations of response shift related to response bias, such as social desirability responding.*

- - Incapacity of verbalising experiences/feelings
  - Irrelevant stimuli

*Code any text that provides examples of stimuli that have resulted in response shift such as the interview questions used or the vignettes.*

- - Other

*Use the “Info box” to provide details as to what other explanations are provided for response shift*

- - None provided

## Table S1: Mixed Methods Appraisal Tool (MMAT), version 2018

Hong QN, Pluye P, Fàbregues S, Bartlett G, Boardman F, Cargo M, Dagenais P, Gagnon M-P, Griffiths F, Nicolau B, O’Cathain A, Rousseau M-C, Vedel I. Mixed Methods Appraisal Tool (MMAT), version 2018. Registration of Copyright (#1148552), Canadian Intellectual Property Office, Industry Canada.

| **Category of study designs** | **Methodological quality criteria** | **Responses** | | | |
| --- | --- | --- | --- | --- | --- |
|  |  | Yes | No | Can’t tell | Comments |
| **Ahmed et al. (2005) [18]** | | | | | |
| Screening questions | S1. Are there clear research questions? |  | X |  | Purpose statement |
|  | S2. Do the collected data allow to address the research questions? | X |  |  |  |
|  | *Further appraisal may not be feasible or appropriate when the answer is ‘No’ or ‘Can’t tell’ to one or both screening questions.* | | | | |
| Mixed methods | 5.1. Is there an adequate rationale for using a mixed methods design to address the research question? | X |  |  |  |
|  | 5.2. Are the different components of the study effectively integrated to answer the research question? | X |  |  |  |
|  | 5.3. Are the outputs of the integration of qualitative and quantitative components adequately interpreted? | X |  |  |  |
|  | 5.4. Are divergences and inconsistencies between quantitative and qualitative results adequately addressed? | X |  |  |  |
|  | 5.5. Do the different components of the study adhere to the quality criteria of each tradition of the methods involved? | X |  |  |  |
| **Category of study designs** | **Methodological quality criteria** | **Responses** | | | |
|  |  | Yes | No | Can’t tell | Comments |
| **Beeken et al. (2011) [23]** | | | | | |
| Screening questions | S1. Are there clear research questions? | X |  |  |  |
|  | S2. Do the collected data allow to address the research questions? | X |  |  |  |
|  | *Further appraisal may not be feasible or appropriate when the answer is ‘No’ or ‘Can’t tell’ to one or both screening questions.* | | | | |
| Qualitative | 1.1. Is the qualitative approach appropriate to answer the research question? | X |  |  |  |
|  | 1.2. Are the qualitative data collection methods adequate to address the research question? | X |  |  |  |
|  | 1.3. Are the findings adequately derived from the data? | X |  |  |  |
|  | 1.4. Is the interpretation of results sufficiently substantiated by data? | X |  |  |  |
|  | 1.5. Is there coherence between qualitative data sources, collection, analysis and interpretation? | X |  |  |  |
| **Category of study designs** | **Methodological quality criteria** | **Responses** | | | |
|  |  | Yes | No | Can’t tell | Comments |
| **Elliott et al. (2014) [27]** | | | | | |
| Screening questions | S1. Are there clear research questions? |  | X |  | Purpose statement |
|  | S2. Do the collected data allow to address the research questions? | X |  |  |  |
|  | *Further appraisal may not be feasible or appropriate when the answer is ‘No’ or ‘Can’t tell’ to one or both screening questions.* | | | | |
| Qualitative | 1.1. Is the qualitative approach appropriate to answer the research question? | X |  |  |  |
|  | 1.2. Are the qualitative data collection methods adequate to address the research question? | X |  |  |  |
|  | 1.3. Are the findings adequately derived from the data? | X |  |  |  |
|  | 1.4. Is the interpretation of results sufficiently substantiated by data? | X |  |  |  |
|  | 1.5. Is there coherence between qualitative data sources, collection, analysis and interpretation? |  | X |  | Unspecified analysis |
| **Category of study designs** | **Methodological quality criteria** | **Responses** | | | |
|  |  | Yes | No | Can’t tell | Comments |
| **Gregory et al. (2005) [28]** | | | | | |
| Screening questions | S1. Are there clear research questions? |  | X |  | Study aim |
|  | S2. Do the collected data allow to address the research questions? | X |  |  |  |
|  | *Further appraisal may not be feasible or appropriate when the answer is ‘No’ or ‘Can’t tell’ to one or both screening questions.* | | | | |
| Qualitative | 1.1. Is the qualitative approach appropriate to answer the research question? | X |  |  |  |
|  | 1.2. Are the qualitative data collection methods adequate to address the research question? | X |  |  |  |
|  | 1.3. Are the findings adequately derived from the data? | X |  |  |  |
|  | 1.4. Is the interpretation of results sufficiently substantiated by data? | X |  |  |  |
|  | 1.5. Is there coherence between qualitative data sources, collection, analysis and interpretation? | X |  |  |  |
| **Category of study designs** | **Methodological quality criteria** | **Responses** | | | |
|  |  | Yes | No | Can’t tell | Comments |
| **King et al. (2019) [24]** | | | | | |
| Screening questions | S1. Are there clear research questions? | X |  |  |  |
|  | S2. Do the collected data allow to address the research questions? | X |  |  |  |
|  | *Further appraisal may not be feasible or appropriate when the answer is ‘No’ or ‘Can’t tell’ to one or both screening questions.* | | | | |
| Qualitative | 1.1. Is the qualitative approach appropriate to answer the research question? | X |  |  |  |
|  | 1.2. Are the qualitative data collection methods adequate to address the research question? | X |  |  |  |
|  | 1.3. Are the findings adequately derived from the data? | X |  |  |  |
|  | 1.4. Is the interpretation of results sufficiently substantiated by data? | X |  |  |  |
|  | 1.5. Is there coherence between qualitative data sources, collection, analysis and interpretation? | X |  |  |  |
| **Category of study designs** | **Methodological quality criteria** | **Responses** | | | |
|  |  | Yes | No | Can’t tell | Comments |
| **Korfage et al. (2006) [20]** | | | | | |
| Screening questions | S1. Are there clear research questions? |  | X |  | Study aim |
|  | S2. Do the collected data allow to address the research questions? | X |  |  |  |
|  | *Further appraisal may not be feasible or appropriate when the answer is ‘No’ or ‘Can’t tell’ to one or both screening questions.* | | | | |
| Qualitative | 1.1. Is the qualitative approach appropriate to answer the research question? | X |  |  |  |
|  | 1.2. Are the qualitative data collection methods adequate to address the research question? | X |  |  |  |
|  | 1.3. Are the findings adequately derived from the data? | X |  |  |  |
|  | 1.4. Is the interpretation of results sufficiently substantiated by data? | X |  |  |  |
|  | 1.5. Is there coherence between qualitative data sources, collection, analysis and interpretation? |  | X |  | Unspecified analysis |
| **Category of study designs** | **Methodological quality criteria** | **Responses** | | | |
|  |  | Yes | No | Can’t tell | Comments |
| **Osborne et al. (2006) [21]** | | | | | |
| Screening questions | S1. Are there clear research questions? |  | X |  | Study aims |
|  | S2. Do the collected data allow to address the research questions? | X |  |  |  |
|  | *Further appraisal may not be feasible or appropriate when the answer is ‘No’ or ‘Can’t tell’ to one or both screening questions.* | | | | |
| Qualitative | 1.1. Is the qualitative approach appropriate to answer the research question? | X |  |  |  |
|  | 1.2. Are the qualitative data collection methods adequate to address the research question? | X |  |  |  |
|  | 1.3. Are the findings adequately derived from the data? | X |  |  |  |
|  | 1.4. Is the interpretation of results sufficiently substantiated by data? | X |  |  |  |
|  | 1.5. Is there coherence between qualitative data sources, collection, analysis and interpretation? |  | X |  | Unspecified analysis |
| **Category of study designs** | **Methodological quality criteria** | **Responses** | | | |
|  |  | Yes | No | Can’t tell | Comments |
| **Rohn et al. (2019) [26]** | | | | | |
| Screening questions | S1. Are there clear research questions? |  | X |  | Purpose statement |
|  | S2. Do the collected data allow to address the research questions? | X |  |  |  |
|  | *Further appraisal may not be feasible or appropriate when the answer is ‘No’ or ‘Can’t tell’ to one or both screening questions.* | | | | |
| Qualitative | 1.1. Is the qualitative approach appropriate to answer the research question? | X |  |  |  |
|  | 1.2. Are the qualitative data collection methods adequate to address the research question? | X |  |  |  |
|  | 1.3. Are the findings adequately derived from the data? | X |  |  |  |
|  | 1.4. Is the interpretation of results sufficiently substantiated by data? | X |  |  |  |
|  | 1.5. Is there coherence between qualitative data sources, collection, analysis and interpretation? | X |  |  |  |
| **Category of study designs** | **Methodological quality criteria** | **Responses** | | | |
|  |  | Yes | No | Can’t tell | Comments |
| **Serdà i Ferrer et al. (2014) [19]** | | | | | |
| Screening questions | S1. Are there clear research questions? |  | X |  | Study aim |
|  | S2. Do the collected data allow to address the research questions? | X |  |  |  |
|  | *Further appraisal may not be feasible or appropriate when the answer is ‘No’ or ‘Can’t tell’ to one or both screening questions.* | | | | |
| Qualitative | 1.1. Is the qualitative approach appropriate to answer the research question? | X |  |  |  |
|  | 1.2. Are the qualitative data collection methods adequate to address the research question? | X |  |  |  |
|  | 1.3. Are the findings adequately derived from the data? | X |  |  |  |
|  | 1.4. Is the interpretation of results sufficiently substantiated by data? | X |  |  |  |
|  | 1.5. Is there coherence between qualitative data sources, collection, analysis and interpretation? | X |  |  |  |
| **Category of study designs** | **Methodological quality criteria** | **Responses** | | | |
|  |  | Yes | No | Can’t tell | Comments |
| **Schwartz & Rapkin (2012) [30]** | | | | | |
| Screening questions | S1. Are there clear research questions? |  | X |  | Hypotheses formulated |
|  | S2. Do the collected data allow to address the research questions? | X |  |  |  |
|  | *Further appraisal may not be feasible or appropriate when the answer is ‘No’ or ‘Can’t tell’ to one or both screening questions.* | | | | |
| Mixed methods | 5.1. Is there an adequate rationale for using a mixed methods design to address the research question? | X |  |  |  |
|  | 5.2. Are the different components of the study effectively integrated to answer the research question? | X |  |  |  |
|  | 5.3. Are the outputs of the integration of qualitative and quantitative components adequately interpreted? | X |  |  |  |
|  | 5.4. Are divergences and inconsistencies between quantitative and qualitative results adequately addressed? |  |  |  | N/A |
|  | 5.5. Do the different components of the study adhere to the quality criteria of each tradition of the methods involved? | X |  |  |  |
| **Category of study designs** | **Methodological quality criteria** | **Responses** | | | |
|  |  | Yes | No | Can’t tell | Comments |
| **Sprangers et al. (2000) [25]** | | | | | |
| Screening questions | S1. Are there clear research questions? |  | X |  | Hypotheses formulated |
|  | S2. Do the collected data allow to address the research questions? | X |  |  |  |
|  | *Further appraisal may not be feasible or appropriate when the answer is ‘No’ or ‘Can’t tell’ to one or both screening questions.* | | | | |
| Mixed methods | 5.1. Is there an adequate rationale for using a mixed methods design to address the research question? | X |  |  |  |
|  | 5.2. Are the different components of the study effectively integrated to answer the research question? | X |  |  |  |
|  | 5.3. Are the outputs of the integration of qualitative and quantitative components adequately interpreted? | X |  |  |  |
|  | 5.4. Are divergences and inconsistencies between quantitative and qualitative results adequately addressed? |  |  |  | N/A |
|  | 5.5. Do the different components of the study adhere to the quality criteria of each tradition of the methods involved? | X |  |  |  |
| **Category of study designs** | **Methodological quality criteria** | **Responses** | | | |
|  |  | Yes | No | Can’t tell | Comments |
| **Taminiau-Bloem et al. (2010) [29]** | | | | | |
| Screening questions | S1. Are there clear research questions? |  | X |  | Study objectives |
|  | S2. Do the collected data allow to address the research questions? | X |  |  |  |
|  | *Further appraisal may not be feasible or appropriate when the answer is ‘No’ or ‘Can’t tell’ to one or both screening questions.* | | | | |
| Qualitative | 1.1. Is the qualitative approach appropriate to answer the research question? | X |  |  |  |
|  | 1.2. Are the qualitative data collection methods adequate to address the research question? | X |  |  |  |
|  | 1.3. Are the findings adequately derived from the data? | X |  |  |  |
|  | 1.4. Is the interpretation of results sufficiently substantiated by data? | X |  |  |  |
|  | 1.5. Is there coherence between qualitative data sources, collection, analysis and interpretation? | X |  |  |  |
| **Category of study designs** | **Methodological quality criteria** | **Responses** | | | |
|  |  | Yes | No | Can’t tell | Comments |
| **Topp et al. (2020) [31]** | | | | | |
| Screening questions | S1. Are there clear research questions? |  | X |  | Purpose statement |
|  | S2. Do the collected data allow to address the research questions? | X |  |  |  |
|  | *Further appraisal may not be feasible or appropriate when the answer is ‘No’ or ‘Can’t tell’ to one or both screening questions.* | | | | |
| Mixed methods | 5.1. Is there an adequate rationale for using a mixed methods design to address the research question? | X |  |  |  |
|  | 5.2. Are the different components of the study effectively integrated to answer the research question? | X |  |  |  |
|  | 5.3. Are the outputs of the integration of qualitative and quantitative components adequately interpreted? | X |  |  |  |
|  | 5.4. Are divergences and inconsistencies between quantitative and qualitative results adequately addressed? | X |  |  |  |
|  | 5.5. Do the different components of the study adhere to the quality criteria of each tradition of the methods involved? | X |  |  |  |
| **Category of study designs** | **Methodological quality criteria** | **Responses** | | | |
|  |  | Yes | No | Can’t tell | Comments |
| **Westerman et al. (2007) [22]** | | | | | |
| Screening questions | S1. Are there clear research questions? |  | X |  | Study aim |
|  | S2. Do the collected data allow to address the research questions? | X |  |  |  |
|  | *Further appraisal may not be feasible or appropriate when the answer is ‘No’ or ‘Can’t tell’ to one or both screening questions.* | | | | |
| Qualitative | 1.1. Is the qualitative approach appropriate to answer the research question? | X |  |  |  |
|  | 1.2. Are the qualitative data collection methods adequate to address the research question? | X |  |  |  |
|  | 1.3. Are the findings adequately derived from the data? | X |  |  |  |
|  | 1.4. Is the interpretation of results sufficiently substantiated by data? | X |  |  |  |
|  | 1.5. Is there coherence between qualitative data sources, collection, analysis and interpretation? | X |  |  |  |

## Table S2: Methods: Description of qualitative response shift studies where a PROM was part of the response shift investigation (Aim 1)

| **Study** | **Qualitative aims, objectives, or research questions pertaining to response shift** | **Definition of response shift used in the study** | **Type of** **response shift studied** | **Sample characteristics, size,**  **sex (male/female) and /or gender (women/men)** | **Qualitative methodology** | **Approach to qualitative analysis** | **Timing of qualitative data collection** | **Recall period used for response shift** | **PROMs used for qualitative inquiry into response shift** | **Interview questions (or other data collection strategies) pertaining to response shift** |
| --- | --- | --- | --- | --- | --- | --- | --- | --- | --- | --- |
| Ahmed et al.  (2005)  [18] | “To assess changes, throughout the first six months post-stroke, in values and conceptualization of HRQOL among individuals as reflected by changes in the content and importance of HRQOL domains generated through the use of the PGI.” (p. 2249) | Sprangers and Schwartz, 1999 [13] | Reprioritization Reconceptualization  General / unspecified response shift | Stroke  (*n=46)*  Larger project included 92 participants, 61% male,  39% female.  “There were no significant differences in sociodemographic variables or physical health between those who were interviewed and those who were not.” (p.2254) | Qualitative approach (unspecified) in mixed methods study | Unspecified analysis | 24 weeks post-stroke | 6 weeks and 24 weeks.  At 24 weeks, participants were shown the answers they provided at 6 weeks and were invited to compare their two sets of responses | PGI | “The following question was asked: ‘If you compare the areas that you just provided to those you gave a few months ago, can you tell me something about why you selected different areas/why certain areas remained the same?’  In addition, a question about significant life events that may have occurred (e.g., marriage, divorce, death in the family) since the last evaluation was asked: ‘Have any events occurred during the last 4 months that have had an influence on your life?’ ” (pp. 2249-2250) |
| Korfage et al.  (2006)  [20] | To explore the “discrepancy between levels of erectile, urinary, or bowel dysfunctions and generic QoL scores. [The authors paid] attention to two mechanisms that could possibly explain the discrepancy: response shift and the insensitivity of generic QoL measures for dysfunctions that prostate cancer patients may perceive after primary treatment.” (p. 913) | Schwartz and Sprangers, 2000 [32] | General / unspecified response shift | Cancer: Prostate cancer patients  (*n=33)*  100% men | Unspecified | Unspecified analysis | 5-6 years (52-80 months) after primary prostate cancer treatment | 5-6 years (52-80 months) | SF36 EQ-5D EQ-5D VAS VAS PCI | “How is your health? Has it been affected by the prostate cancer treatment? If yes, how? How is your QOL? Has it been affected by the prostate cancer treatment?” (p. 913) |
| Osborne et al.  (2006)  [21] | “To determine the extent to which response shift occurs, and to determine if a paper-based questionnaire, the HEI-Q Perspective, can accurately identify the presence of response shift.” (p. 459) | Sprangers and Schwartz, 1999 [13] | Recalibration  General / unspecified response shift | Arthritis and other chronic diseases  (*n=39)*  13% male.  87% female | Unspecified | Unspecified analysis | Questionnaires sent 10 months post programme completion and interviews 4 weeks post questionnaire completion | 4 weeks | HEI-Q | “Can you tell me more about that answer?  If necessary, 2 additional prompts were used:  How did you come to see it that way?  Is there anything else you would like to say about that?” (pp. 459-460) |
| Rohn et al.  (2019)  [26] | “To expand understanding of QOL as it is experienced; to gain greater insight into individuals’ QOL definitions, appraisals and adaptations following spinal cord injury.” (p. 470) | Schwartz and Sprangers, 2000 [32] | Recalibration  Reconceptualization  General / unspecified response shift | Spinal cord injury  (*n=40)*  80% male,  20% female | Content analysis | Content analysis | Unspecified | Unspecified | PROMIS-36 Bladder and Bowel Behavioral Management Questionnaire SCI-specific measures of QOL BBTI | “What kind of complications have you had related to your bladder? To your bowel?  How do you feel about your relationship/experience with your caregiver?  In general, to what extent has the loss of bladder and bowel control impacted the relationships with people around you?  Can you tell me how bladder and bowel dysfunction impacts your ability to have intimate and sexual relationships? To what extent has loss of bladder and bowel control impacted relationships with family or household members? Professional relationships? Community involvement?  In terms of your future goals, what do you hope to be doing [years] down the road?  What areas of your life do you find most satisfying? Least satisfying?  What areas pertaining to QOL are most important to you right now?” (p. 477) |
| Serdà i Ferrer et al. (2014)  [19] | “To identify QoL response shift mechanisms in men with prostate cancer, describe the relationship between side effects and QoL, and assess the magnitude and direction of response shift after primary treatment.” (p. 34) | Sprangers and Schwartz, 1999 [13]  &  Schwartz and Sprangers, 2000 [32] | Recalibration Reprioritization  Reconceptualization | Cancer: Prostate cancer patients  (*n=66)*  100% men | Grounded theory | Constant comparative analysis | Three time periods: Baseline, 16-week post treatment, Two weeks after P2 | 16 and 18 weeks  posttreatment | FACT-P FACIT-Fatigue | “1. How are you feeling? How is your health?  2. Has your health been affected by the treatment? If yes, please explain.  3. How would you describe your current QOL?  4. Would you say that your QOL has changed since the day the prostate cancer was diagnosed? If yes, please explain.  5. Which are the most important spheres/domains/dimensions of your life?  6. How do they contribute to maintaining your QOL?” (p. 35) |
| **Schwartz & Rapkin**  **(2012)**  **[30]** | “The purpose of the present work is to investigate the thentest-minus-pretest discrepancy score in terms of underlying cognitive appraisal processes, and its relationship with antecedents and catalysts.” (p. 382) | Sprangers and Schwartz, 1999 [13]  &  Schwartz and Sprangers, 1999 [14] | Recalibration | HIV/AIDS  *(n=521)*  53% male,  47% female | Qualitative approach (unspecified) in mixed methods study | Content analysis | Baseline at 6 weeks post-enrolment, and at 6 months post-baseline | 6 months post baseline | SF-36 Version 2  QOL Appraisal Profile | "Data from the 6-month time point included the following additional questions: (1) items asking if the person recalled the answer s/he gave to the global QOL at baseline, and if so, to provide this answer (to investigate recall accuracy); (2) a thentest item assessing global QOL; (3) an item asking if the way that the person thinks and feels about his/her overall health has changed since baseline, and if so, how (open-ended text response)." (p. 383) |
| Sprangers et al.  (2000)  [25] | “To examine whether response shift occurred, resulting from changes in internal standards of measurement.” (p. 140) | Sprangers and Schwartz, 1999 [14] | Recalibration | Cancer (breast, lung and prostate)  *(n=74)*  Larger project included 105 participants at baseline,  40% male,  60% female | Qualitative approach (unspecified) in mixed methods study | Unspecified analysis  Interviews rated and response shift “defined to be revealed when (1) patients report a difference between their responses to the pretest and thentest, which may either result from initial overrating or underrating, and (2) patients explicitly refer to a re-evaluation at the thentest of pretest levels of functioning.” (p. 142) | Interviews after thentest | 4-7 weeks  Pretest completed after first session of radiotherapy.Postest and thentest completed after the last session of radiotherapy.Thentest recall: one week prior to first radiotherapy | 3 fatigue items of the EORTC QLQ-30, version 1.0  MFI-20 | “The interviewer asked patients to compare their answers to the posttest with those to the pretest. Permissible questions were whether the answers were generally in agreement or in disagreement as long as they were posed in this order. Interviewers were instructed to provide open, nonjudgmental probes (e.g., C*an you amplify your answer? Can you give an example?)* Interviewers were further instructed to elicit spontaneous or invited comparisons but not to use leading questions to address fatigue. The interviewers and the patients were blinded to the results of the pretreatment assessment.” (p. 141) |
| Taminiau-Bloem et al.  (2010)  [29] | “To examine whether the content of each distinct cognitive process underlying QoL appraisal is (dis)similar over time and whether patterns of (dis)similarity can be discerned across and within patients and/or items.” (p. 1) | Rapkin and Schwartz, 2004 [33]  (also making use of Tourangeau, Rips and Rasinski’s model [34] | Recalibration Reprioritization  Reconceptualization | Cancer  (*n=50)*  48% men,  52% women | TSTI combining cognitive think-aloud interviewing and verbal probing techniques | Qualitative analysis scheme based on the cognitive process models of Tourangeau et al. and Rapkin and Schwartz for the subsequent coding of patients’ cognitive processes | Baseline interviews same day as simulator appoint-ment or the first radio-therapy treatment; Follow-up interviews conducted on last day of radio-therapy | 27-82 days | 7 items of the EORTC QLQ-C30 | “What does [target construct in item, e.g., QOL] mean to you? Can you tell me how you came to think of [aspect mentioned by respondent]? Did you compare yourself to someone or something? How did you arrive at your response? Can you tell me why you choose the selected response category?” (p. 2) |
| Topp et al.  (2020)  [31] | 1) “Outline the development and evaluation of the anchoring vignettes.”  2) “Describe how anchoring vignettes were used in a patient sample and address challenges and lessons learned regarding the anchoring vignette approach.” (p. 2150) | Sprangers and Schwartz, 1999 [13] | General / unspecified response shift | Patients with psoriasis or multiple sclerosis  (*n=100)*  48% male,  52% female | Qualitative approach (unspecified) in mixed methods study | Unspecified analysis | Baseline and 26 months later | Unspecified | SF-12 | “During both interviews, patients assessed HRQOL of 16 hypothetical individuals on the SF-12 questionnaire (two vignettes for each of the eight domains of the SF-12).” (p. 2149) |
| Westerman et al.  (2007)  [22] | “To search for response shift type explanations to account for counter-intuitive ﬁndings in QoL measurement.” (p. 853) | Sprangers and Schwartz, 1999 [13] | Recalibration Reprioritization  Reconceptualization | Small-cell lung cancer  (*n=23)*  48% male,  52% female | Other: exploratory, longitudinal multiple-case study | Triangulated mind maps of think aloud data, PROM scores, and response shift explanation (recalibration, reprioritization,  reconceptualization) | T1: Start of chemotherapy T2: 4 weeks after T1 T3: 10 days after chemotherapy completion T4: 6 weeks later | The period between the current and previous assessment | EORTC QLQ-C30 | No questions listed |

BBTI = Bladder and bowel treatment methods; EORTC QLQ-C30 = European organization for research and treatment of cancer – Quality of life questionnaire 30; EQ5D VAS = EQ-5D Visual analogue scale; FACT-P = Functional assessment of cancer therapy – Prostate; FACIT-Fatigue = Functional assessment of chronic illness therapy – Fatigue; HEI-Q = Health education impact questionnaire; HRQOL = Health related quality of life;

QOL = Quality of life; MFI-20 = Multidimensional Fatigue Index 20; PCI = Prostate cancer index; PGI = Patient generated index; PROMIS-36 = patient-reported outcomes measurement information system; SF12 = Short form 12; SF36 = Short form 36; SCI = Spinal cord injury; TSTI = Three-step test Interview.

## Table S3: Methods: Description of qualitative response shift studies when response shift was investigated with qualitative methods (Aim 1)

| **Study** | **Qualitative aims, objectives, or research questions pertaining to response shift** | **Definition of response shift used in the study** | **Type of response shift studied** | **Sample characteristics, size,**  **sex (male/female) and /or gender (women/men)** | **Qualitative methodology** | **Approach to qualitative analysis** | **Timing of qualitative data collection** | **Recall period used for response shift** | **Interview questions (or other data collection strategies) pertaining to response shift** |
| --- | --- | --- | --- | --- | --- | --- | --- | --- | --- |
| Beeken et al.  (2011)  [23] | “To explore the role of psychosocial variables in adjustment to compromised HRQOL following haematopoietic stem cell transplant, from the theoretical basis of the response-shift model.” (p. 153) | Sprangers and Schwartz, 1999 [13] | Recalibration Reprioritization  Reconceptualization | Haematopoietic stem cell transplant patients  *(n= 28)*  46% men,  54% women | Unspecified | Template analysis | Unspecified | Compared to pre-transplant | “Questions to explore the existence of response shift included the following: 1) What does HRQOL mean and how has it changed? (reconceptualization). 2) How they would describe their current HRQOL, how this compared to their HRQOL pre-transplant, whether they would have viewed their HRQOL in the same way pre-transplant if asked at the time rather than in retrospect, whether a good day (physically/socially/ emotionally/cognitively) meant different things to them now as opposed to pre-transplant? (recalibration).  3) Whether different things were important to them now? (changing values).” (pp. 154-155) |
| Elliott et al.  (2014)  [27] | “To improve the general understanding of elders’ lives while living with dialysis and to provide new insights into the values and decision-making of older dialysis patients and their families.” (p. 1498) | Sprangers and Schwartz, 1999 [13]  &  Schwartz and Sprangers, 1999 [14] | Recalibration Reprioritization  Reconceptualization | 31 interviews with *n=20* people living with dialysis and *n=11* family members.  Sex/gender not reported for family members.  Sex reported for 27 people living with dialysis  (56% male,  44% female)  but not for the 20/27 who participated in interviews. | Unspecified | Unspecified analysis | Unspecified | Unspecified | “1. Tell us about your experience with kidney disease and dialysis. Probes: How has kidney disease affected your life; your mood, outlook, and attitudes; your family and friendships? Describe what has been important in your experience  2. In general, how have you managed up to now? Probes: Tell us about your personal outlook or values; the role of family and social relationships; religion and spirituality. What sources of support have you had?  3. What do you think has affected the course of your kidney disease? Probes: Tell us about your role, the roles of your family, your care providers, and nature or fate in the course of your illness  4. Tell us about your decision to continue dialysis/forego dialysis Probes: How does this fit into your life (story)? What has been most important? How long have you been thinking about this? Who else has been influential in your decision? 5. Tell us about any conversations you have had about advance care planning. Probes: What about healthcare directives, living wills, and advance directives? What are your wishes now? Who have you talked with? Who is your proxy/decision-maker?  6. Is there anything else you would like to tell us about? Probes: Are there issues with family burden or family conflict; finances; religion; faith; treatment burden; symptoms; control?  7. What advice do you have for improving the support of people with CKF? Probes: Do you have any suggestions in regard to the care of the patient or the family?” (p. 1499) |
| Gregory et al.  (2005)  [28] | “To explore the relationship between ill health and QOL, this project aimed to ﬁnd out how assessments of oral HRQOL vary between and change within individuals.” (p. 1861) | Sprangers and Schwartz, 1999 [13] | Recalibration  Reconceptualization | Oral health: People with socially noticeable broken, decayed or missing teeth who were or were not seeking dental treatment  *(n= 20).*  Sex and/or gender not reported | Grounded theory | Integration of systems theory with the grounded theory method | 2nd interview took place 3 months after first | 3 months | “In the first interview, participants were asked about daily lives, their challenges and hopes for the future to obtain a feeling for their general expectations and the context in which they talked about oral health. Photographs of people with varying degrees of oral health and disease were introduced during the interview to stimulate observations without pre-structuring the interview. The second interview took place up to three months later to see if the way participants talked about oral health had changed. More directive prompts were used including dental leaflets and artefacts such as dental floss, mouthwash, a dental mirror and probe. Participants were asked what QOL meant to them and how they thought it might relate to oral health.” (p. 1861) |
| King et al.  (2019)  [24] | “The purpose of this study was to investigate occupational therapists’ and physical therapists’ perspectives on the process of change that occurs in clients during rehabilitation.” (p. 355)  Research question: “How are the concepts of the process of change used by occupational therapists and physiotherapists related to the concepts of change described in transformative learning or response shift?” (p. 357) | Sprangers and Schwartz, 1999 [13]  &  Schwartz and Sprangers, 1999 [14] | Reprioritization  Reconceptualization  General / unspecified response shift | Occupational therapists & physiotherapists  *(n= 23)*  Sex and/or gender not reported | Interpretative description | Thematic analysis and content analysis | Unspecified | Unspecified | “The focus group discussion began with the participants being presented with a hypothetical clinical scenario, which described the changes that a client, Julian, who had a spinal cord injury, had undergone during rehabilitation.” (p. 358)  “Clinical scenario - Julian  •What do you think is happening regarding Julian’s story?  •How do you think Julian changed during rehabilitation?  •What do you think he changed? Prompt: his perspective? His outlook?  •What would you call the change that occurs with Julian?  •How would you document Julian’s change in your chart?  •How would you measure it?  •How would you describe change in your clients? What do you observe?  •How do you know when personal change has occurred with your clients?” (p. 366) |

CKF = chronic kidney failure; HRQOL = Health related quality of life; QOL = Quality of life.
